# Identification of key immune cells infiltrated in lung adenocarcinoma microenvironment and their related long noncoding RNA

**DOI:** 10.1016/j.isci.2024.109220

**Published:** 2024-02-15

**Authors:** Kai Wang, Tao Yan, Deyu Guo, Shijie Sun, Yong Liu, Qiang Liu, Guanghui Wang, Jingyu Chen, Jiajun Du

**Affiliations:** 1 Institute of Oncology, Shandong Provincial Hospital, Shandong University, Jinan, China; 2 Department of Healthcare Respiratory Medicine, Shandong Provincial Hospital, Shandong University, Jinan, China; 3 Lung Transplantation Center, The Affiliated Wuxi People’s Hospital of Nanjing Medical University, Wuxi People’s Hospital, Wuxi Medical Center, Nanjing Medical University, Wuxi 214023, China; 4 Institute of Oncology, Shandong Provincial Hospital Affiliated to Shandong First Medical University, Jinan, China; 5 Department of Thoracic Surgery, Shandong Provincial Hospital, Shandong University, Jinan, China

**Keywords:** classification Description, microenvironment, molecular biology, immunology, cancer, machine learning

## Abstract

LncRNA associated with immune cell infiltration in tumor microenvironment (TME) may be a potential therapeutic target for lung adenocarcinoma. We established a machine learning (ML) model based on 3896 samples characterized by the degree of immune cell infiltration, and further screened the key lncRNA. *In vitro* experiments were applied to validate the prediction. Treg is the key immune cell in the TME of lung adenocarcinoma, and the degree of infiltration is negatively correlated with the prognosis. PCBP1-AS1 may affect the infiltration of Tregs by regulating the TGF-β pathway, which is a potential predictor of clinical response to immunotherapy. PCBP1-AS1 regulates cell proliferation, cell cycle, invasion, migration, and apoptosis in lung adenocarcinoma. The results of clinical sample staining and *in vitro* experiments showed that PCBP1-AS1 was negatively correlated with Treg infiltration and TGF-β expression. Tregs and related lncRNA PCBP1-AS1 can be used as targets for the diagnosis and treatment of lung adenocarcinoma.

## Highlights

• Treg cell was screened as tumor-associated immune cell by machine learning
• Multiple visualization toolkits interpret model prediction criteria
• Treg cell-associated lncRNA was screened as PCBP1-AS1 by machine learning method
• PCBP1-AS1 regulates the TGF-β pathway and response to immunotherapy

## Graphical abstract

**Figure:**
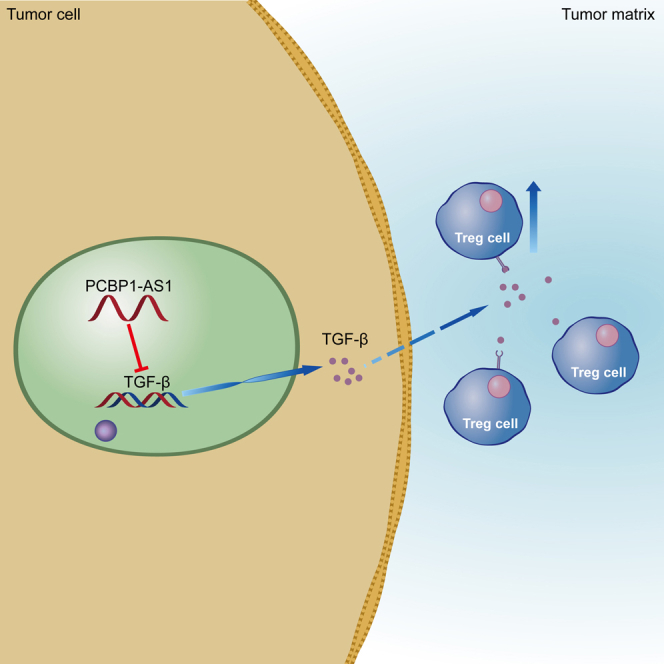

## Introduction

The incidence of lung cancer ranks second, and the mortality rate ranks first globally.^1^ Non-small cell lung cancer (NSCLC) accounts for approximately 80%–85% of lung tumors.^2^ Lung adenocarcinoma is the most common pathological type of NSCLC, which accounts for approximately 40% of all lung tumors.^3^ Owing to the clinical application of immunotherapy, the prognosis of patients with lung cancer has improved significantly. A previous study showed that the median overall survival (OS) time of patients with lung cancer was 20.2 months in the atezolizumab group and 13.1 months in the chemotherapy group.^4^ Unfortunately, 60% of patients are resistant to immunotherapy or can only partially respond to immunotherapy.^5^ Due to the unavailability of more effective treatment, the five-year survival rate of patients with lung cancer is still only 23%.^1^ Therefore, new biomarkers and therapeutic targets for lung cancer should be found.

Tumor microenvironment (TME) is a highly structured environment containing cancer cells that are surrounded by different non-malignant cell types, embedded in a changed, vascularized extracellular matrix. TME contains a rich variety of immune cells, cancer-associated fibroblasts (CAFs), endothelial cells (ECs), and other cell types.^6^ The degree of infiltration of different immune cells in the TME greatly affects immunotherapy.^7^^,^^8^^,^^9^^,^^10^ TME is regulated by tumor cell metabolism,^11^ tumor stroma, including tumor cells in the TME, can produce TGF-β, which can induce the differentiation and infiltration of Treg cells.^12^^,^^13^ Treg cells cooperate with tumor cells to escape tumor-specific immune responses.^14^^,^^15^^,^^16^^,^^17^^,^^18^ Tregs are a unique type of inhibitory CD4^+^T cells, which act as major negative regulators of inflammation and immunity in many biological environments.^19^^,^^20^ The best-characterized Treg subsets are defined by the expression of coreceptor CD4, cytokine receptor CD25, and transcription factor Foxp3 (encoded by X-linked genes).^21^ Tregs can kill effector T cells via granzyme and perforin or inhibit effector T cells by promoting adenosine production. Furthermore, Tregs can competitively consume IL-2 with effector T cells and inhibit the survival of effector T cells.^22^ Local depletion of Tregs can significantly alleviate lung cancer, whereas the high infiltration of Tregs implies a poor immune response.^22^ Therefore, Tregs are considered an important target for cancer immunotherapy.

LncRNA exhibits several complex characteristics and various functions, making it a useful therapeutic target. This form of RNA is > 200 bp long and plays a major role in the metabolism, proliferation, and apoptosis of tumors and other crucial cancer-associated processes.^23^ Many types of lncRNA provide researchers with several opportunities to discover valuable therapeutic targets or biomarkers that can indicate the progression of the disease.^24^^,^^25^^,^^26^

The diversified machine learning (ML) algorithm is a data analysis method, which develops an algorithm to predict the results by learning from the data and can help find therapeutic targets for lung cancer.^27^ The clinical application of ML ranges from diagnosis to prediction and has been used in various clinical fields.^28^^,^^29^^,^^30^^,^^31^ For predicting the prognosis of critically ill patients, the results of the ML method are better than those obtained by using traditional logical regression and Cox regression analyses. However, because of the black-box nature of ML algorithms, ML models are difficult to be applied in the field of medicine due to a lack of interpretability.^32^ To analyze the results of the ML model, we used four ML visualization toolkits, namely SHAP, PDPBox, ELI5, and InterpretML, to gain an in-depth understanding of the contribution of different features to the model and predict the complex relationship between features and tags. Furthermore, we have also provided an intuitive explanation, which is helpful to completely understand how a developed model can make specific prediction.

In this study, we collected the cohort of lung adenocarcinoma from The Cancer Genome Atlas Program (TCGA), Genotype-Tissue Expression (GTEx), and Gene Expression Omnibus (GEO) databases and determined the effect of immune cell infiltration on the formation of lung adenocarcinoma using an ML model and visual analysis. We then screened lncRNA with the highest effect on Treg infiltration using the ML model. A clinical prognostic model of adenocarcinoma with 4-lncRNA expression was established. Owing to the size of the differential expression and the clinical and immune system significance, a lncRNA, poly r (C) binding protein 1 antisense (PCBP1-AS1), was considered to be particularly important. We elucidated the mechanism underlying PCBP1-AS1 by proliferation assay, EdU assay, wound healing assay, transwell assay, and apoptotic assay, and predicted the response of immunotherapy to investigate the clinical value of PCBP1-AS1. Lastly, we used our lung adenocarcinoma cohort to verify the results of the bioinformatics analysis.

## Results

### Screening regulatory T cells as the key immune cells infiltrated in lung adenocarcinoma using the machine learning model

**Figure 1:**
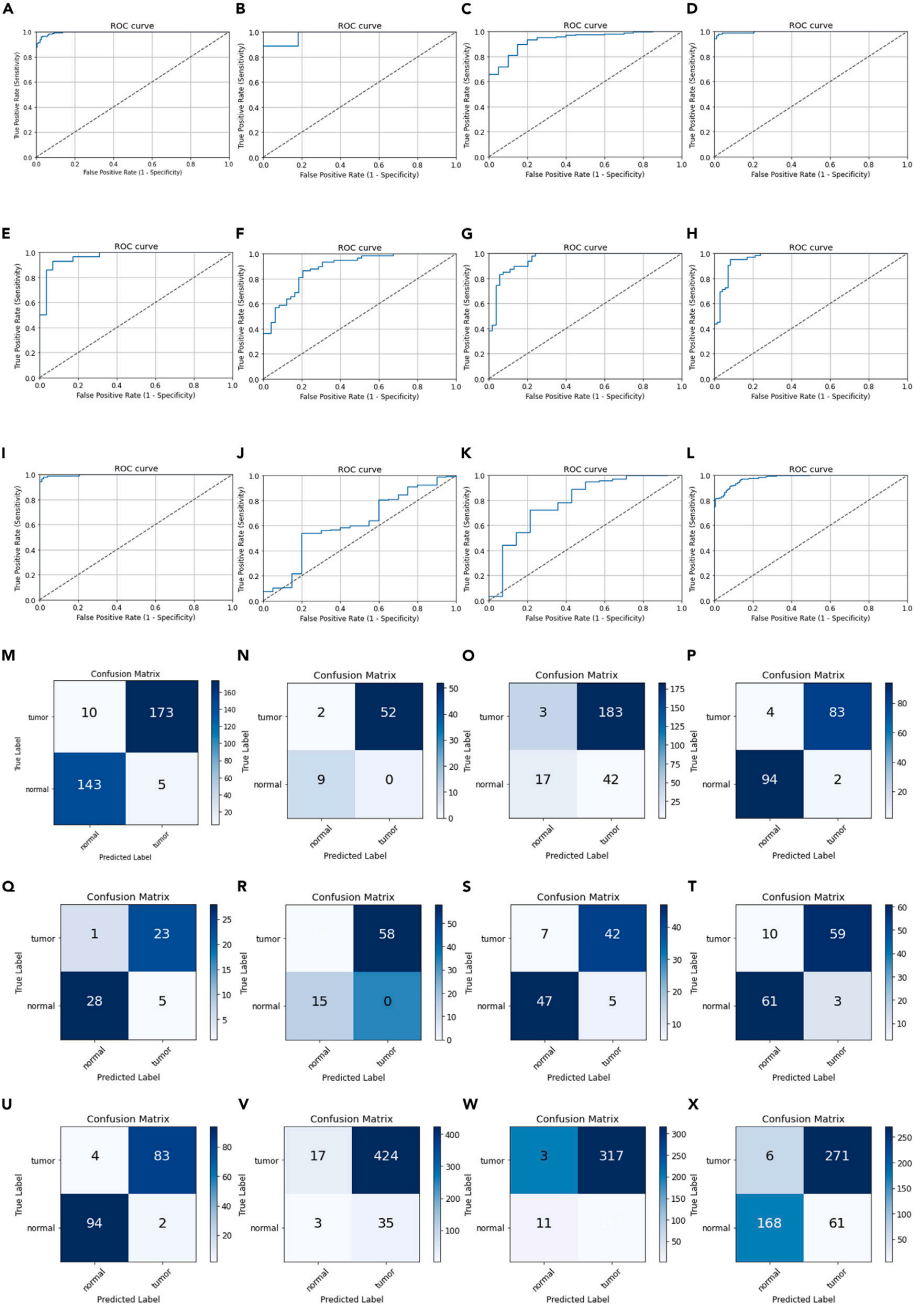
Prediction effect of immune infiltration machine learning model (A) The ROC curve of the internal validation the training set. (B–L) The ROC curve of the external validation of the validation set. (M) The confusion matrix of the internal verification of the training set. (N–X) The confusion matrix of the external validation of the validation set.

A total of 3896 lung adenocarcinoma and normal tissues were collected from the TCGA, GTEx, and GEO datasets and divided into 12 groups under the premise of a 1:1 ratio of normal and tumor tissues as much as possible (Table S2). The Cibersort package was used to detect the degree of immune cell infiltration in each group. Considering 22 types of immune cells as characteristics and tumor and normal tissues as tags, group 1 was modeled using XGBoost. The prediction efficacy of the model was internally verified using the ROC curve and confusion matrix (Figures 1A and 1M) and externally verified based on the observations from the remaining 11 groups (Figures 1B–1L and 1N–1X).

**Figure 2:**
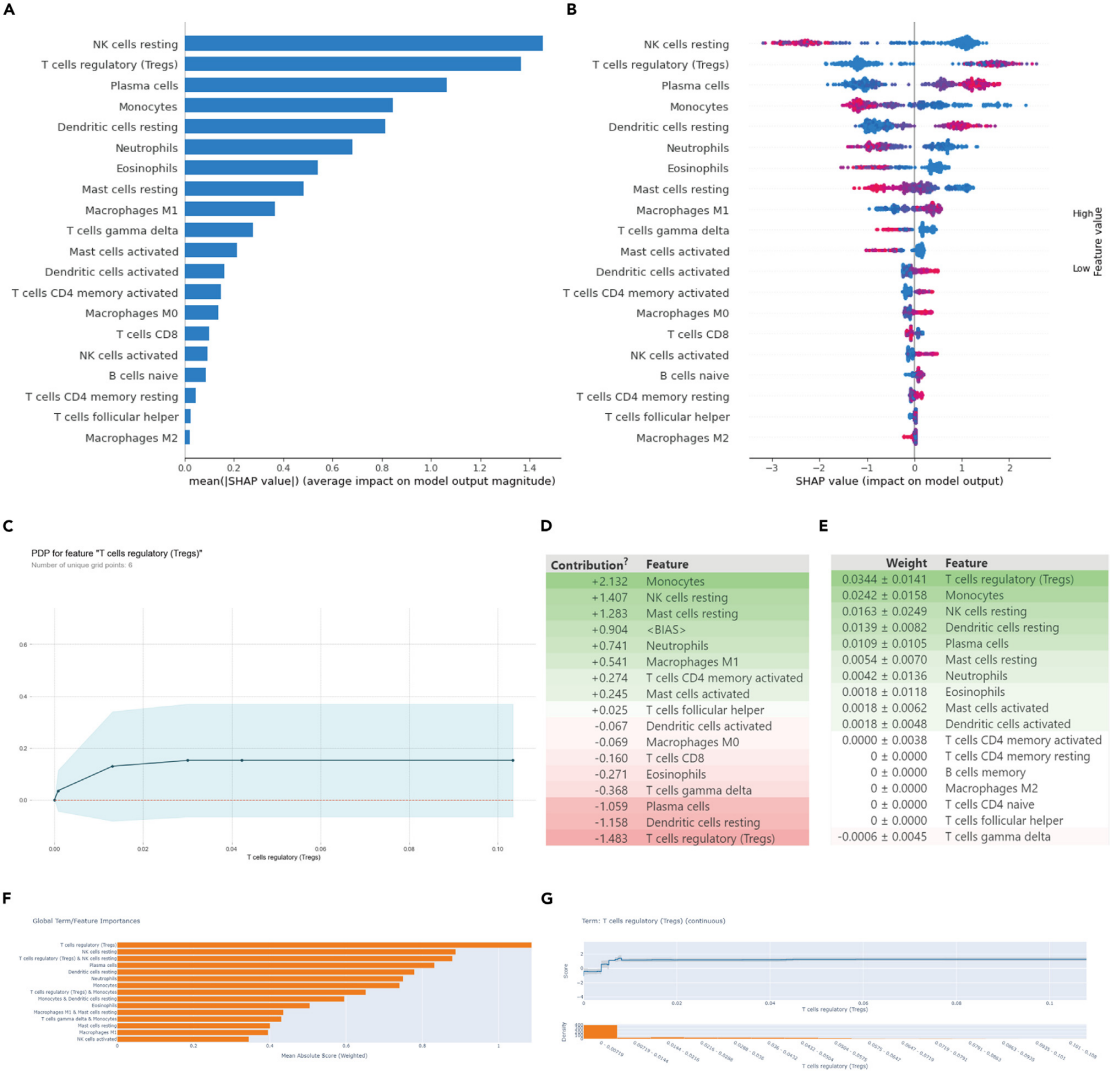
Visual Analysis of immune infiltration Machine Learning Model (A) Ranking of model feature contribution in SHAP. (B) The influence of different features on model prediction in SHAP. (C) PDPBox shows the effect of changes in Tregs infiltration on model prediction. (D) Feature contribution information predicted by ELI5. (E) Score of permutation importance function on feature contribution in ELI5. (F) Summary of InterpretML to model feature importance. (G) InterpretML shows the effect of Tregs infiltration degree on model prediction. Influence of model prediction results.

To explore the effect of the expression of different immune cells on the prediction results of the model, we used four ML visual analysis toolkits, such as SHAP, PDPBox, ELI5, and InterpretML, and analyzed the importance of features. SHAP values showed the top 20 important features (Figure 2A). Moreover, we performed SHAP correlation analysis to study the effect of a single variable on the final results of XGBoost and found that increasing Treg infiltration was associated with a greater probability of tumorigenesis (Figure 2B). To further determine the relationship between the degree of Treg infiltration and the results predicted by the model, we used PDPBox and visualized the role of Treg cells. We found that as the degree of Treg infiltration increased from 0 to 0.03, the probability of a sample being a tumor increased (Figure 2C). To verify the findings of SHAP and PDPBox, we used the Eli5 toolkit, which showed that the degree of Treg infiltration was the most important feature and the weight was about 0.0344 (Figures 2D and 2E). Next, we used InterpretML and comprehensively interpreted the model, which was consistent with previous results. The most important feature was the degree of Treg infiltration (Figure 2F). Furthermore, we analyzed the interaction between Tregs and tags, which was consistent with the results obtained using PDPbox. With the increase in Treg infiltration, the probability of a sample being a tumor increased (Figure 2G). The results showed that Tregs had the greatest contribution to model prediction.

### SHapley additive exPlanations model predicts tumor proportion

In order to verify the extended application value of the visualization model, the SHAP values of the training set of the ML model that screened Treg were used for PCA unsupervised clustering, and the samples were divided into two groups (Figure S1A). By comparing the proportion of normal and tumor tissues in the two groups of samples, we defined them as the high-risk group and the low-risk group (Figure S1B), and set them as the labels of the ML model to predict the sample groups of 11 validation sets, respectively. We found that in all validation sets, the proportion of tumor samples in the high-risk group was significantly higher than that in the low-risk group (Figures S1C–S1M). This indicated that the model established by SHAP values had a clear classification effect and significance for clinical transformation.

### Screening of long noncoding RNAs related to regulatory T cells infiltration using the machine learning model

**Figure 3:**
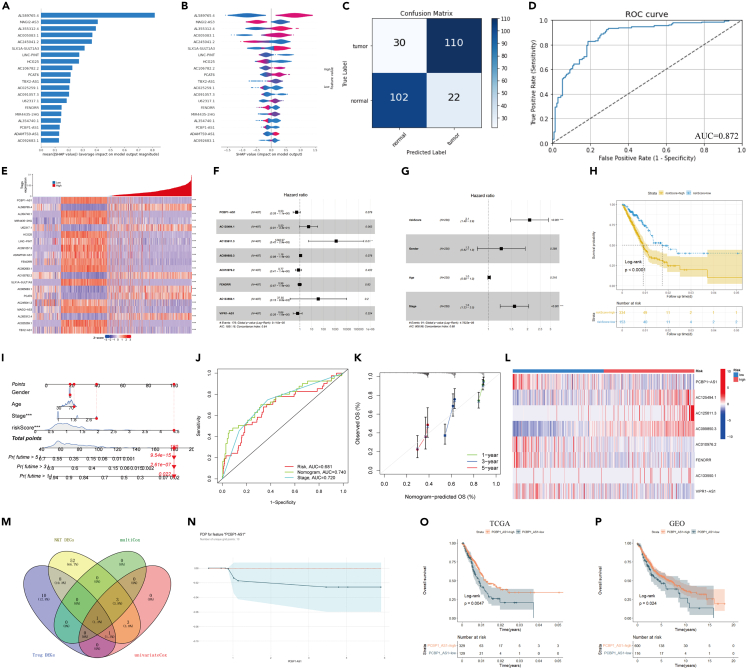
Screening of Treg related lncRNAs (A) SHAP shows the top 20 feature contribution lncRNA. (B) SHAP shows the influence of different features on the prediction effect of the model. (C) Confusion matrix verified within the training set. (D) ROC curve verified within the training set. (E) The heatmap of the lncRNA with the top 20 feature contribution in the Tregs high and low infiltration group. (F) Multivariate Cox regression analysis of Treg related lncRNAs. (G) Multivariate Cox regression Model with lncRNAs Risk Score, gender, age and stage. (H) Survival analysis of high and low risk groups based on lncRNA Risk Score. (I) Construction of a nomogram model of based on multivariate Cox regression model with lncRNAs Risk Score, gender, age and stage. (J) ROC curve of the nomogram model. (K) Calibration curves for the nomogram model for 1, 3, and 5 years (L) Heatmap of characteristic genes based on lncRNAs Risk score in high and low risk groups. (M) Intersection of differential genes in tumor and paracancerous tissues, marker genes in univariate and multivariate Cox regression models and Treg related lncRNAs. (N) PDPBox shows the effect of PCBP1-AS1 expression on the infiltration of Tregs in machine learning. (O) Survival analysis of PCBP1-AS1 in TCGA lung adenocarcinoma dataset. (P) Survival analysis of PCBP1-AS1 in GEO united lung adenocarcinoma datasets.

To explore lncRNAs related to Treg infiltration, we used Cibersort and analyzed the immune infiltration of the GTEx and TCGA samples, followed by sample division into high- and low-infiltration groups according to the values of Cibersort Treg infiltration scores. We analyzed differences between the groups and obtained 147 differentially expressed lncRNAs (Table S3). Considering these lncRNAs as features and high and low Treg infiltration as tags, we modeled the groups using XGBoost. To understand the effect of different lncRNAs on the degree of Treg infiltration, we used SHAP and other interpretable packages for the analysis. SHAP values showed the top 20 important features (Figure 3A). Furthermore, SHAP correlation analysis was performed to understand the effect of a single variable on the final result of XGBoost (Figure 3B). To evaluate the prediction accuracy of the ML model, we used the ROC curve and confusion matrix and analyzed the model (Figures 3C and 3D). The heatmap showed the relationship between the top 20 lncRNAs and Treg infiltration (Figure 3E).

### Differential expression of key long noncoding RNAs related to prognosis in lung adenocarcinoma

The analysis of the integrated data from GTEx and TCGA indicated 68 differently expressed lncRNAs between normal and carcinoma tissues. 5 of them were upregulated, whereas 63 were downregulated (Table S4). To establish a prognostic model, 487 eligible patients with lung adenocarcinoma were selected. According to univariate and multivariate Cox regression analyses, eight lncRNAs with independent prognostic significance were found. Detailed statistical information is provided in Table S5.

The hazard ratios of the eight lncRNAs are presented in a forest map plotted using the R packages survival and survminer (Figure 3F). For this prognostic model, the p value was 9.12 × 10^−5^ (<0.001) according to a log rank test, and the C index was 0.64. The lncRNAs risk score was calculated using the R package "survival," which contains PCBP1-AS1, AC125494.1, AC125611.3, and AC099850.3. We obtained the also lncRNAs Risk Score based on Cox analysis: lncRNAs Risk Score = −0.67338∗PCBP1-AS1 FPKM +1.76070∗AC125494.1 FPKM +7.66041∗AC125611.3 FPKM +0.21155∗AC099850.3 FPKM, with the threshold value = 0.9889 (equally divided into high-risk and low-risk groups, lncRNAs Risk Score≥0.9889 as the high risk group). According to risk scores, the patients were divided into low-risk (n = 244) and high-risk (n = 243) groups. A summary of the differential expression of genes in the high-risk and low-risk groups is presented in Figure 3L. Then, the multivariate Cox regression analysis performed using factors such as lncRNAs risk score, gender, age, and stage also showed that lncRNAs risk score was an independent risk factor (Figure 3G). A significant difference in OS was found between the high- and low-risk groups; patients with lung adenocarcinoma in the high-risk group showed poorer OS rates. (p < 0.0001, Figure 3H). We further constructed a nomogram based on the results of multivariate Cox analysis, and the results showed that the risk score had the significant predictive value for the prognosis of patients (Figures 3I–3K). The AUC of the overall nomogram, variables stage and lncRNAs risk score were all greater than 0.68, indicating that the prediction efficiency was satisfactory.

### Poly r (C) binding protein 1 antisense is closely related to regulatory T cells infiltration in tumor microenvironment and the prognosis of patients with lung adenocarcinoma

To analyze differences between normal and tumor lncRNAs, we intersected the top 20 lncRNAs mentioned above, including Treg infiltration-related and survival-related lncRNAs screened by univariate and multivariate Cox regression analyses, and PCBP1-AS1 was obtained (Figure 3M). We explored the specific regulation of PCBP1-AS1 on Treg infiltration using PDPBox and found that with increasing PCBP1-AS1 expression, the degree of Treg infiltration decreased significantly (Figure 3N). Kaplan–Meier curves were plotted to express lung adenocarcinoma microarray data derived from four GEO datasets (GSE30219, GSE37745, GSE50081, and GSE72094) and lung adenocarcinoma sequencing data from TCGA. We found that patients with high PCBP1-AS1 expression showed a better prognosis (Figures 3O and 3P).

### Enrichment analysis based on poly r (C) binding protein 1 antisense expression

**Figure 4:**
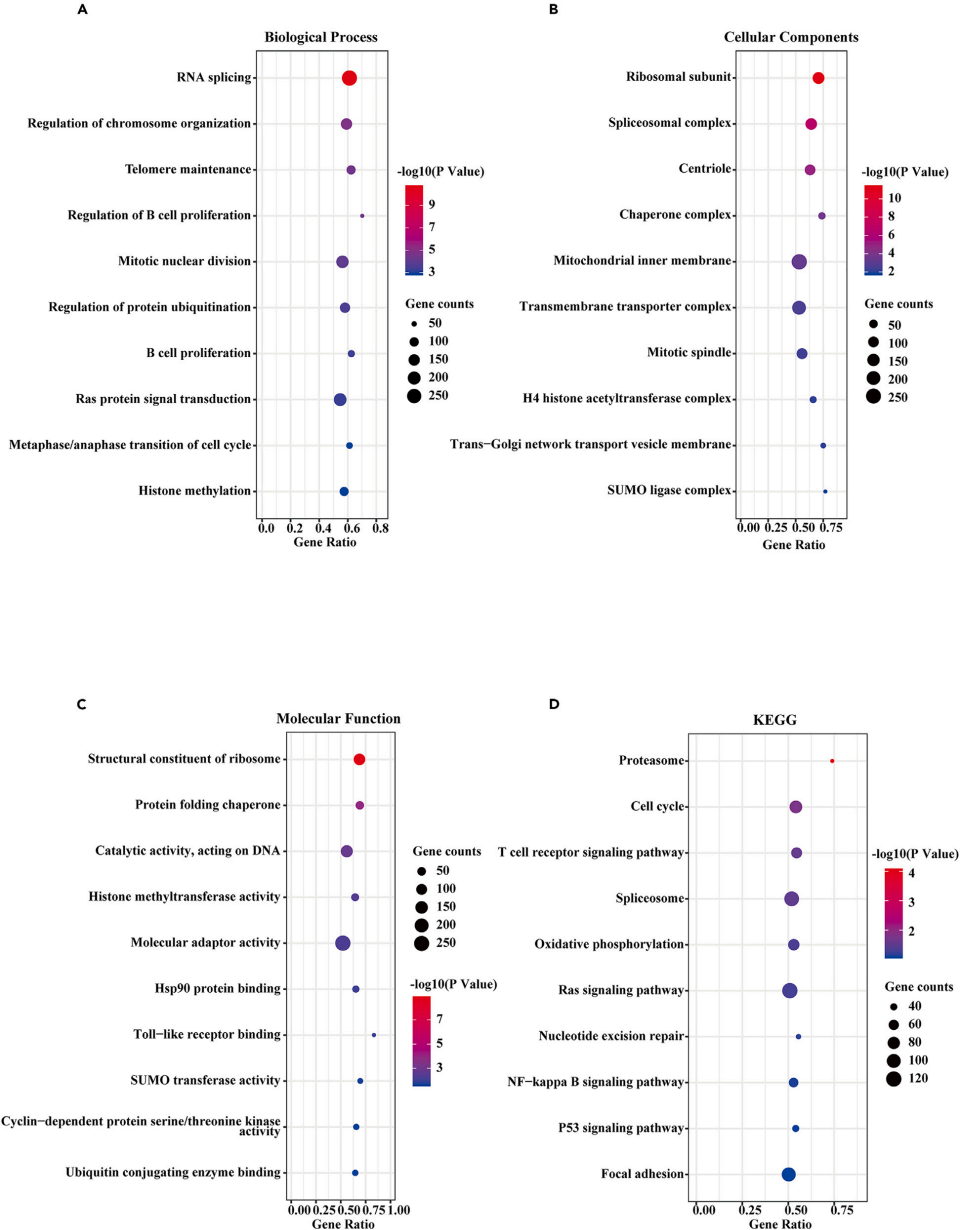
Functional enrichment analysis of PCBP1-AS1 in TCGA LUAD dataset (A) Biological process analysis. (B) Cellular components analysis. (C) Molecular function. (D) KEGG pathway analysis.

**Figure 5:**
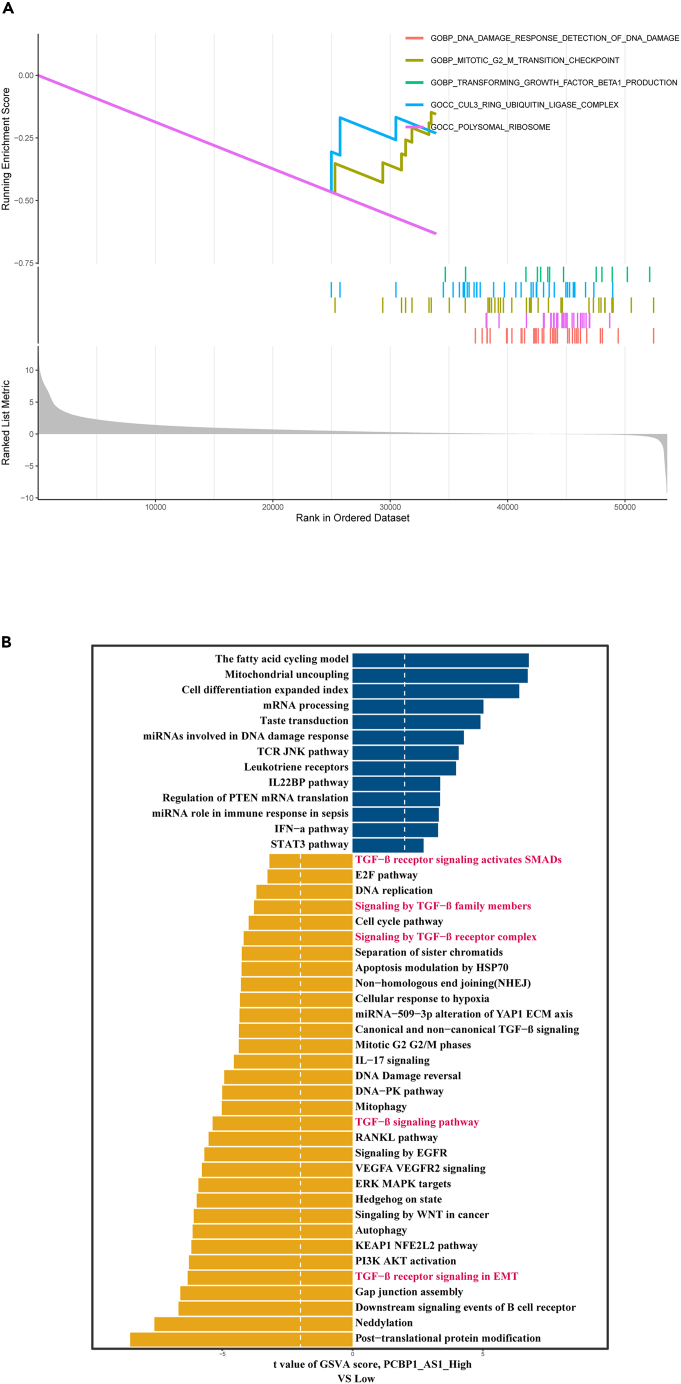
Exploring the mechanism of PCBP1-AS1 in shaping tumor microenvironment in TCGA LUAD dataset (A) GSEA analysis of GO terms. (B) GSVA analysis.

We first used TRlnc ^33^ and LncSEA^34^ to explore the mechanism of the transcriptional regulation of PCBP1-AS1 in lung adenocarcinoma and its possible biological role, the results showed that PCBP1-AS1 mainly functioned through RNA-RNA interaction and RNA-protein interaction, and mainly regulated tumor immunity (Figure S2). Then we analyzed the differential genes of PCBP1-AS1 in the TCGA samples by performing GO and KEGG enrichment analyses. The GO enrichment results (Figures 4A–4C) included biological processes (BPs), cell composition (CC), and molecular functions (MFs). Cross-gene enriched BPs were mainly involved in RNA splicing and the regulation of chromosome organization. CC was mainly involved in the ribosomal subunit, spliceosomal complex, and centriole. MFs were mainly related to the structural constituent of the ribosome, protein folding chaperone, and catalytic activity, acting on DNA. The KEGG enrichment results showed that proteasome, cell cycle, and T cell receptor signaling pathways were mainly involved (Figure 4D). The results of GSEA showed that PCBP1-AS1 expression was related to DNA damage response, polysome ribosome, mitotic G2/M transition checkpoint, and TGF-β production (Figure 5A). To further investigate functional characteristics, we divided the datasets of lung adenocarcinoma in TCGA into two groups according to PCBP1-AS1 expression and performed GSVA enrichment analysis. The results showed that PCBP1-AS1 expression was negatively correlated with TGF-β related pathways, including TGF-β signaling pathway, TGF-β receptor signaling in epithelial-mesenchymal transition (EMT), signaling by the TGF-β receptor, signaling by the TGF-β receptor complex, and TGF-β receptor signaling activating downstream SMADs (Figure 5B).

### Correlation analysis of poly r (C) binding protein 1 antisense and its effect on regulatory T cell infiltration

**Figure 6:**
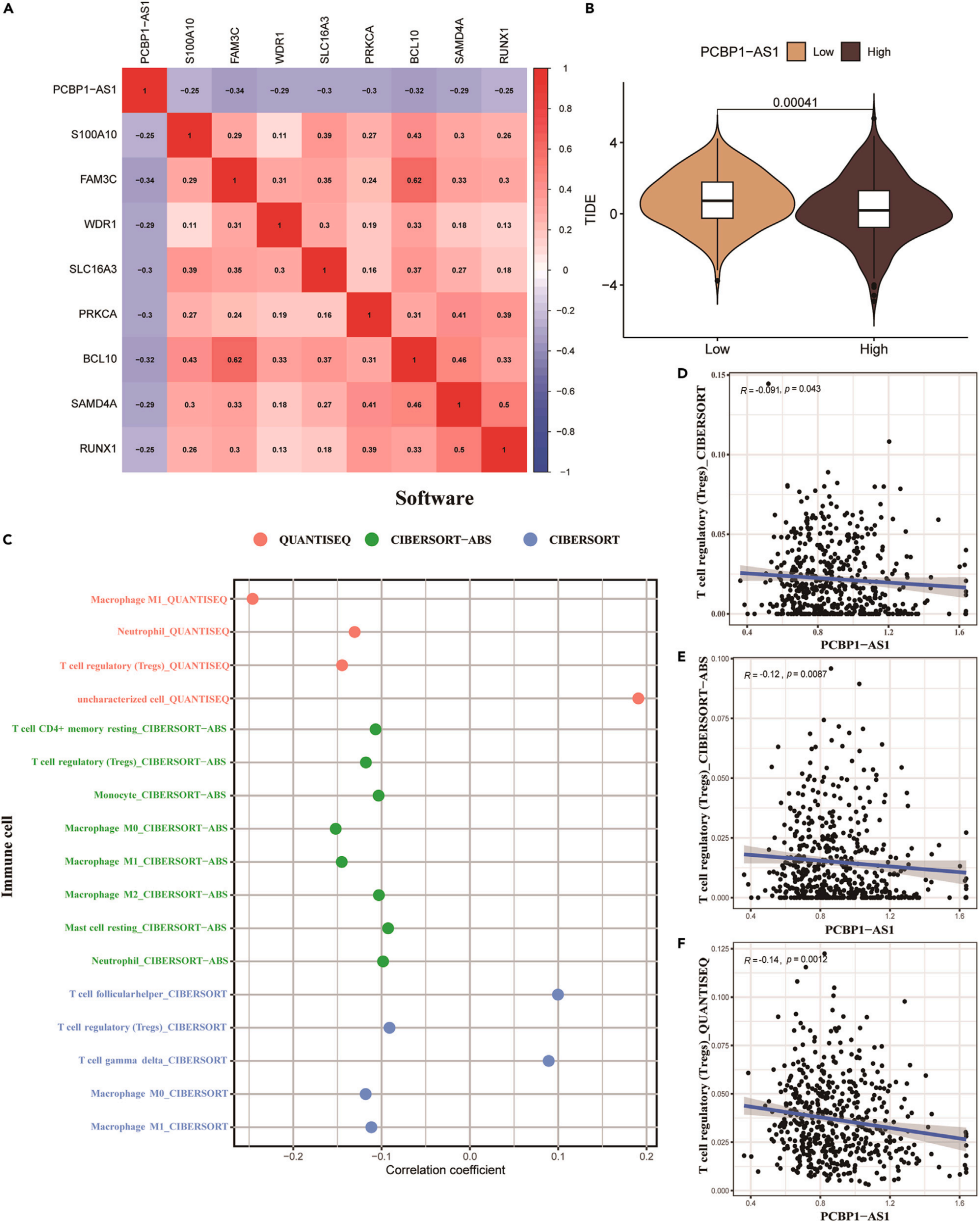
Analysis of PCBP1-AS1 expression on the benefits of immunotherapy (A) Correlation of PCBP1-AS1 and PCBP1-AS1 significantly related molecules by Pearson correlation analysis. (B) The influence of PCBP1-AS1 on immunotherapy efficacy predicted by TIDE analysis. (C) The correlation of PCBP1-AS1 and immune cells infiltrated. (D–F) The correlation of PCBP1-AS1 expression and regulatory T cells via CIBERSORT-ABS, CIBERSORT and QUANTISEQ.

The results of the correlation analysis showed that PCBP1-AS1 was significantly negatively correlated with RUNX1, the upstream regulator of the TGF-β pathway (Figure 6A). Moreover, PCBP1-AS1 also showed a significant negative correlation with FAM3C, BCL10, SLC16A3, WDR1, and other key molecules of tumor malignant behavior.

The infiltration analysis of immune cells showed that PCBP1-AS1 was negatively correlated with Treg cells, macrophages, and neutrophils, but positively correlated with helper T cells and γδT cells (Figure 6C). In order to investigate the immune infiltration of PCBP1-AS1 and regulatory T cells (Treg cells), three QUANTISEQ, CIBERSORT-ABS, and CIBERSORT algorithms were used to evaluate the immune cell infiltration (Figures 6D–6F). The results showed that the expression of PCBP1-AS1 and Treg cells was negatively correlated (p value < 0.05).

### Prediction and validation of immunotherapy efficacy in high and low poly r (C) binding protein 1 antisense expression groups

**Figure 7:**
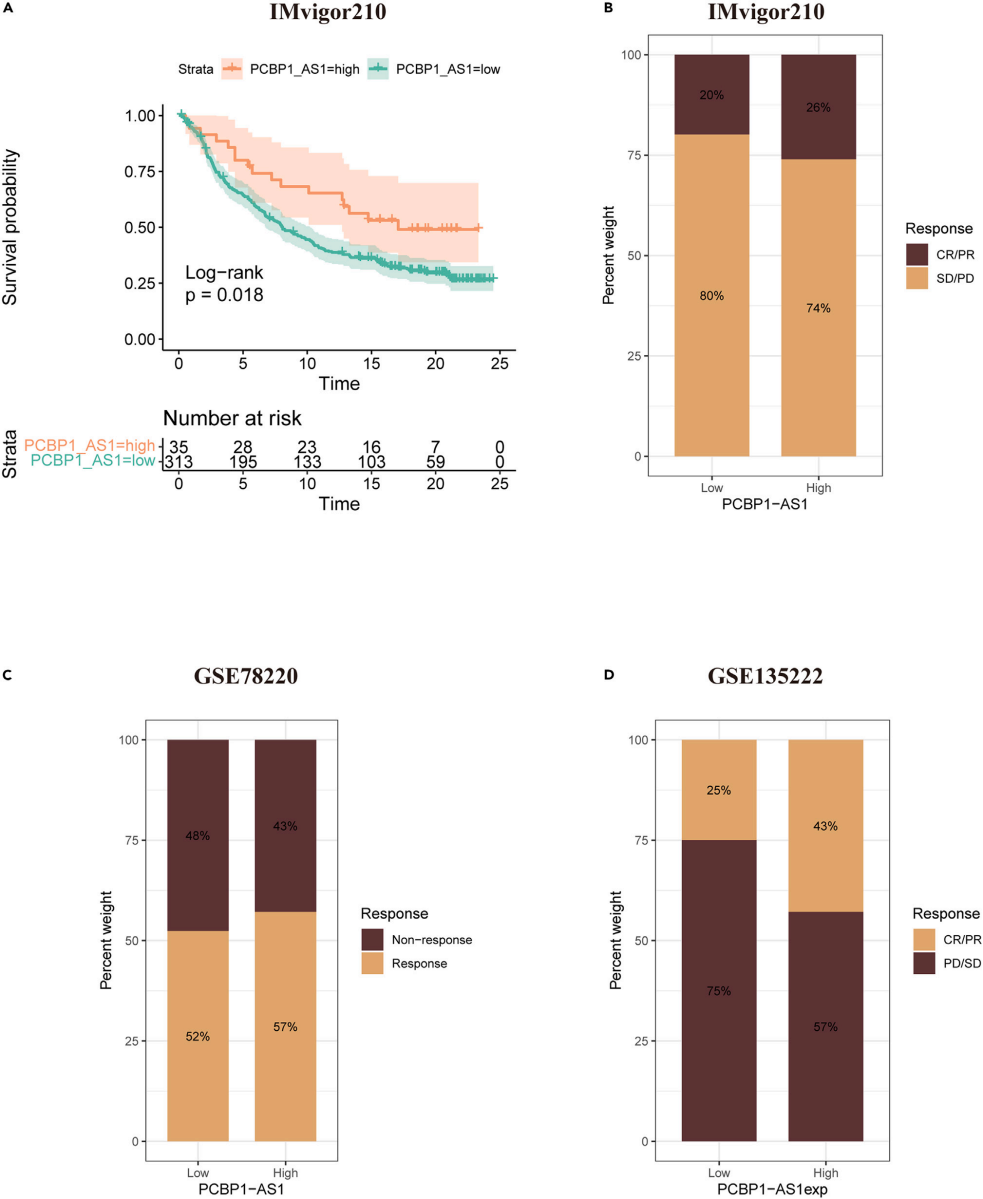
Real-world immunotherapy data Validation Response to anti-PD-L1 therapy. (A) Difference on overall survival (OS) between high and low PCBP1-AS1 groups in IMvigor210 cohort. Differences on response to immunotherapy between high and low PCBP1-AS1 groups in (B) IMvigor210, (C) GSE78220 and (D) GSE135222 cohorts.

To further investigate the correlation between PCBP1-AS1 expression and immunotherapy efficacy, we calculated the TIDE score. Patients with low PCBP1-AS1 expression showed higher TIDE scores (Figure 6B), indicating the worse efficacy of immunotherapy, including ICIs. Kaplan-Meier analysis showed that patients with the high expression of PCBP1-AS1 had a significant OS advantage over those with the low expression of PCBP1-AS1 in the IMvigor210 immunotherapy cohort (p = 0.018) (Figure 7A). To further validate the immunotherapy effect of PCBP1-AS1, we applied immunotherapy cohorts for three different cancer types (IMvigor210 bladder cancer, GSE78220 melanoma, and GSE135222 NSCLC). The proportion of immunotherapy response, or CR/PR, in patients with the high expression of PCBP1-AS1 was higher than that in patients with low expression, which indicated that the PCBP1-AS1 overexpression group responded better to immunotherapy (Figures 7B–7D).

### Poly r (C) binding protein 1 antisense regulates lung adenocarcinoma proliferation and cell cycle

**Figure 8:**
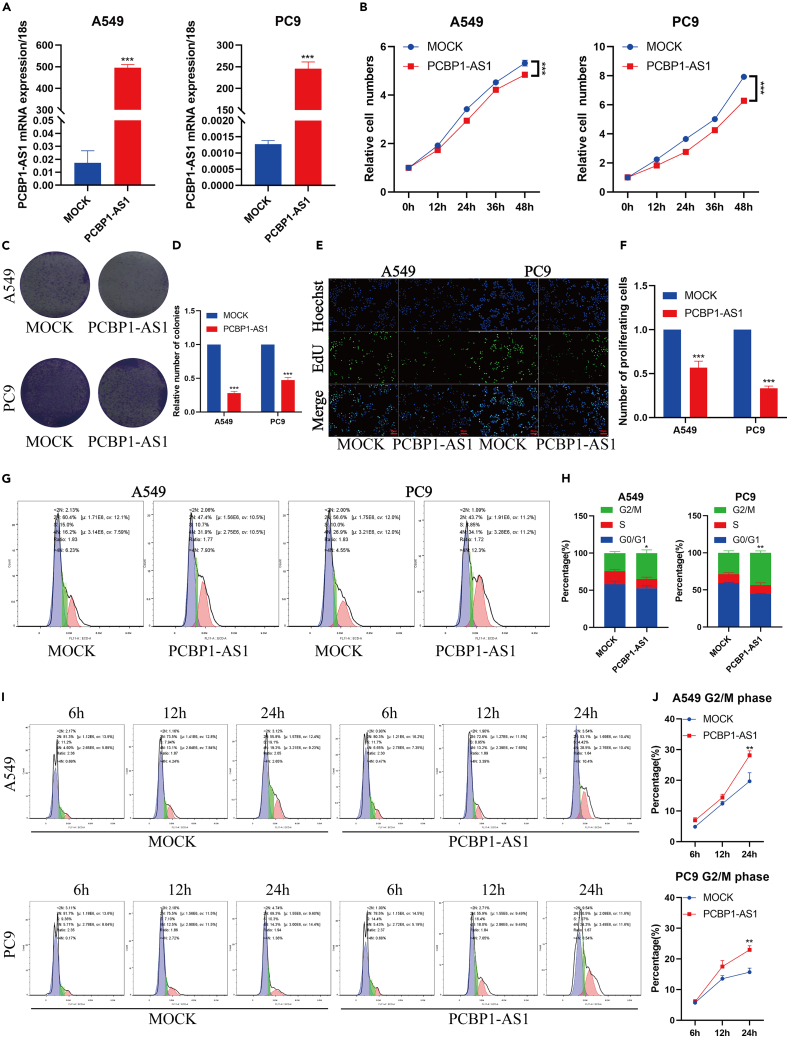
PCBP1-AS1 inhibits the proliferation and induces cell-cycle arrest (A) A549 and PC9 cells overexpressing PCBP1-AS1. (B) SRB staining assay was used to detect cell proliferation. (C) Overexpression of PCBP1-AS1 inhibits clone formation, which is reflected in the (D) number of colony formation. (E and F) EdU assay demonstrated the over-expressing of PCBP1-AS1 inhibited the DNA replication of lung adenocarcinoma cells. (G and H) Flow cytometry indicated the over-expression of PCBP1-AS1 resulted in cell-cycle arrest at G2/M phase in both A549 and PC9 cells compared with control group. (I and J) After 24 h of serum-free culture, full culture medium was recovered, compared with the control group, the proportion of G2/M phase cells in the group with the overexpression of PCBP1-AS1 increased with the prolongation of collection time. ^∗^p < 0.05, ^∗∗^p < 0.01, ^∗∗∗^p < 0.001. n = 3 independently treated cultures, p values were calculated using an unpaired Student’s *t* test and mean ± s.e.m. is presented.

To further verify the role of PCBP1-AS1 in lung adenocarcinoma, a series of behavioral experiments were performed. We found that the cell proliferation rate of A549 and PC9 was significantly slower after the overexpression of PCBP1-AS1 (Figures 8A and 8B). The colony formation assay showed that the number of colony formation cells was significantly reduced after the overexpression of PCBP1-AS1 (Figures 8C and 8D). Subsequently, we performed an EdU assay, and the results showed that PCBP1-AS1 also had an inhibitory effect on DNA replication in lung adenocarcinoma cells (Figures 8E and 8F). To further explore how PCBP1-AS1 inhibits cell proliferation and DNA replication, we performed the cell cycle assay and found that PCBP1-AS1 had a significant cell-cycle arrest effect on lung adenocarcinoma cells. After the overexpression of PCBP1-AS1, lung adenocarcinoma cells were arrested in the G2/M phase (Figures 8G and 8H). Lung adenocarcinoma cells were arrested at the G0/G1 phase by starvation treatment for 24 h, and then cultured in serum. The cell cycle was detected at different time points, the results also showed that lung adenocarcinoma cells were arrested in G2/M phase (Figures 8I and 8J), which was consistent with the previous enrichment analysis.

### Poly r (C) binding protein 1 antisense regulates EMT and apoptosis in lung adenocarcinoma cells

**Figure 9:**
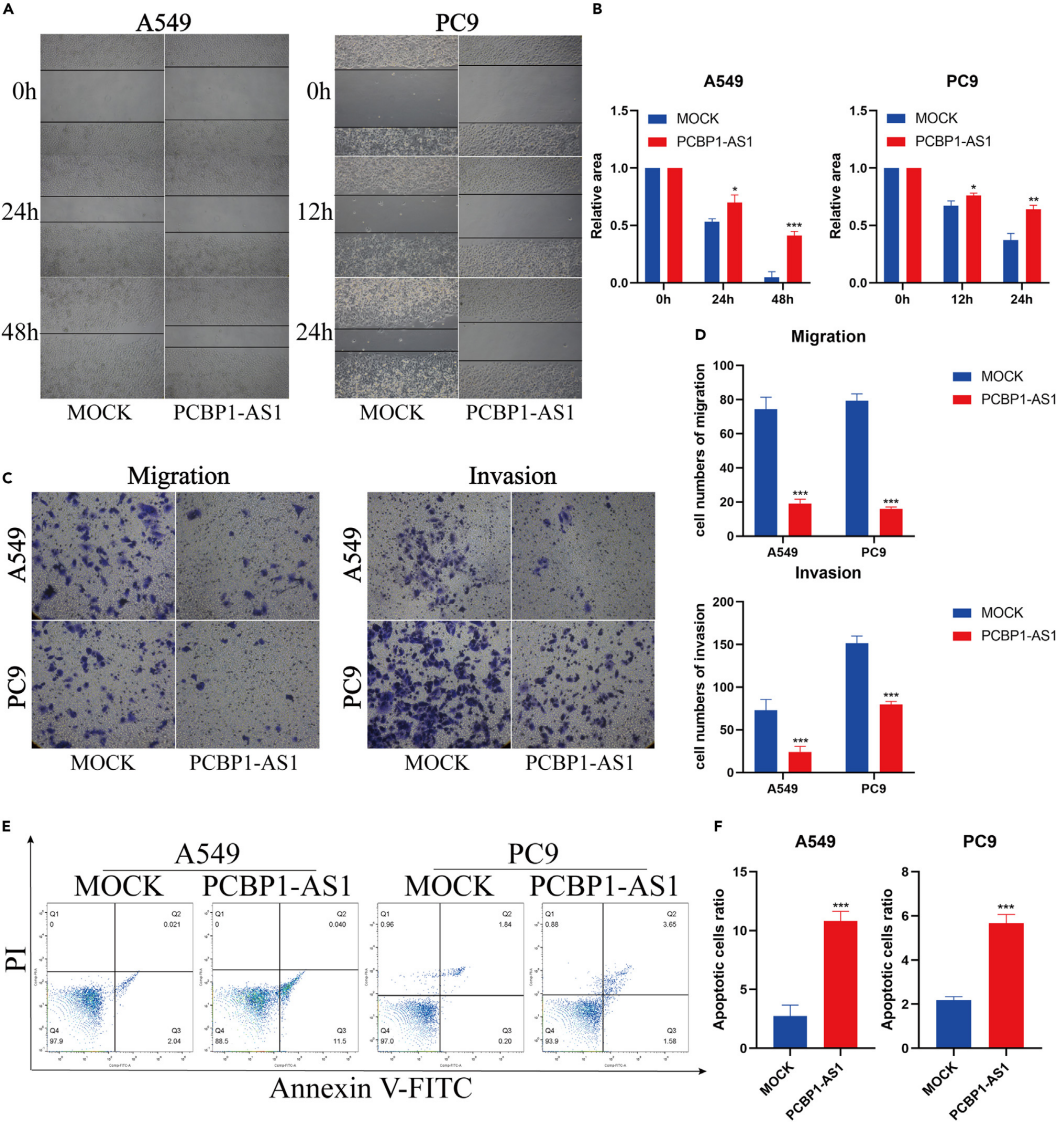
PCBP1-AS1 inhibits migration and invasion, and promotes the apoptosis of lung adenocarcinoma cells (A and B) A wound-healing experiment was used to analyze the migration of overexpressing PCBP1-AS1 cells and control cells. (C and D) Transwell assays were used to analyze the migration and invasion of overexpressing PCBP1-AS1 cells and control cells. (E and F) Annexin V-FITC/PI staining showed that PCBP1-AS1 could promote apoptosis of lung adenocarcinoma cells. ^∗^p < 0.05, ^∗∗^p < 0.01, ^∗∗∗^p < 0.001. n = 3 independently treated cultures, p values were calculated using an unpaired Student’s *t* test and mean ± s.e.m. is presented.

We further explored other biological roles of PCBP1-AS1 in lung adenocarcinoma. Through wound healing experiments, we found that the migration ability of lung adenocarcinoma cells was significantly inhibited after the overexpression of PCBP1-AS1 (Figures 9A and 9B). A Transwell assay also showed that PCBP1-AS1 could inhibit the invasion and migration ability of lung adenocarcinoma cells (Figures 9C and 9D). The level of cell apoptosis was detected by flow cytometry, and the results showed that the degree of cell apoptosis was increased after the overexpression of PCBP1-AS1 (Figures 9E and 9F).

### Poly r (C) binding protein 1 antisense expression was negatively correlated with regulatory T cells infiltration

**Figure 10:**
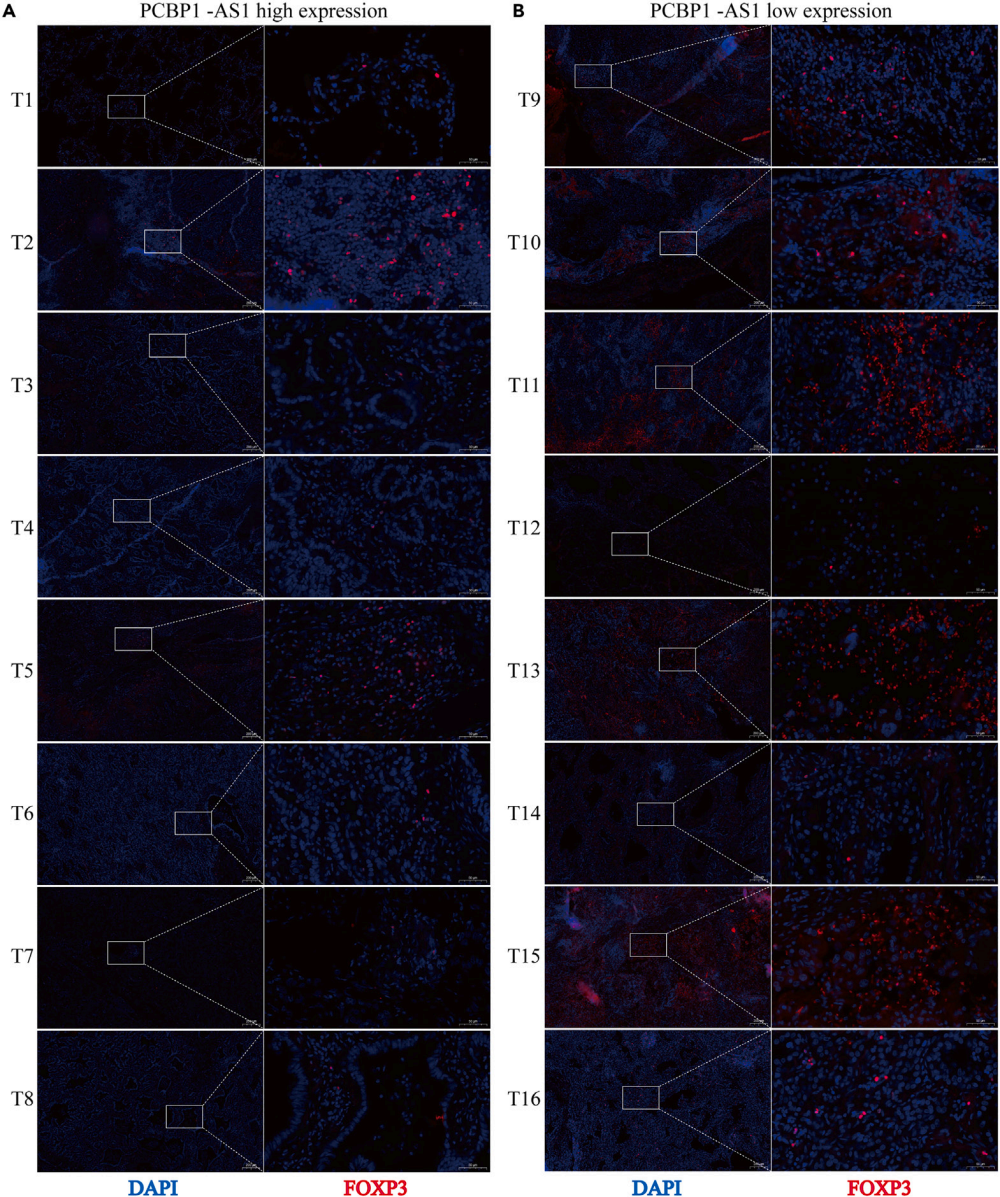
PCBP1-AS1 expression was negatively correlated with Treg infiltration (A) FOXP3 expression in the high PCBP1-AS1 expression group. (B) FOXP3 expression in the low PCBP1-AS1 expression group.

To verify the correlation between PCBP1-AS1 expression and Treg infiltration in lung adenocarcinoma, RT-qPCR, and immunofluorescence assays were performed on 16 lung adenocarcinoma samples. We equally divided the samples into PCBP1-AS1 high and low expression groups according to the expression level of PCBP1-AS1 through RT-qPCR results. The expression level of FOXP3, a marker of Treg cells, was detected by immunofluorescence. We found that the expression level of FOXP3 was significantly lower in the PCBP1-AS1 high expression group than in the PCBP1-AS1 low expression group (Figures 10A and 10B), indicating that PCBP1-AS1 expression was negatively correlated with the degree of Treg cell infiltration in lung adenocarcinoma.

### Validation of the clinical significance of poly r (C) binding protein 1 antisense in the Shandong Provincial Hospital (SPH) cohort

**Figure 11:**
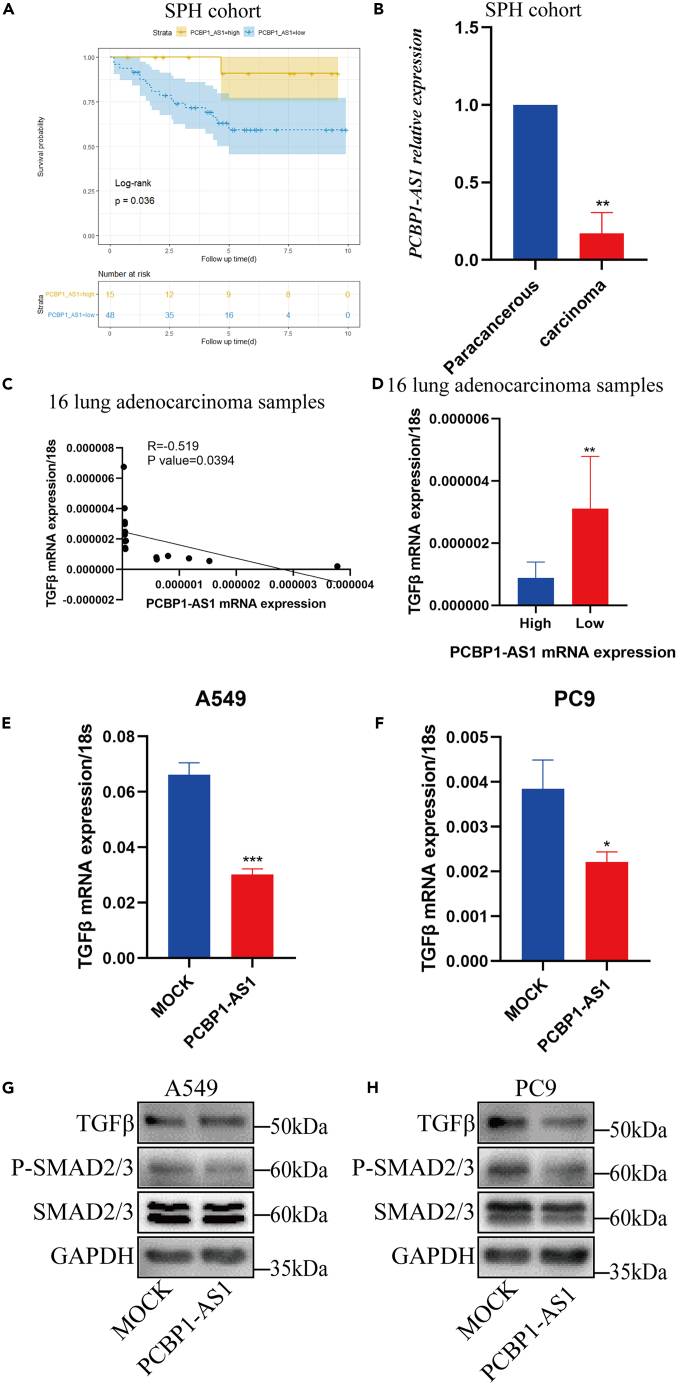
PCBP1-AS1 expression was negatively correlated with TGF-β expression and related pathways (A) Kaplan-Meier curve showed overall survival difference of 63 patients with lung adenocarcinoma divided based on expression level of PCBP1-AS1. (B) Relative expression level of PCBP1-AS1 between tumor samples and their matched para-cancerous tissues. (C and D) PCBP1-AS1 expression was negatively correlated with TGF-β mRNA expression in 16 lung adenocarcinoma samples. (E and F) Over-expression of PCBP1-AS1 reduced TGF-β expression in lung adenocarcinoma cells. (G and H) PCBP1-AS1 expression was negatively correlated with the activation of TGF-β pathways. ^∗^p < 0.05, ^∗∗^p < 0.01, ^∗∗∗^p < 0.001. n = 3 independently treated cultures, p values were calculated using an unpaired Student’s *t* test and mean ± s.e.m. is presented.

The prognostic information of the 63 patients supported the claim that patients with high PCBP1-AS1 expression showed a significantly better prognosis (p = 0.036, Figure 11A). The relevant clinical baseline data is provided in Table 1. Moreover, considering its status as a prognostic protective factor, PCBP1-AS1 expression in neoplasms was much lower than that in adjacent tissues (Figure 11B).

**Table 1:** Clinicopathological characteristics of patients with LUAD

Characteristics	number	proportion
**Gender**		

female	29	46.00%
male	34	54.00%

**Age**		

<65	45	71.40%
≥65	18	28.60%

**Location**		

left	27	42.90%
right	36	57.10%

**Smoking history**		

no	41	65.10%
yes	22	34.90%

**T stage**		

T1	38	60.30%
T2	22	34.90%
T3	3	4.80%

**N stage**		

N0	41	65.10%
N1	15	23.80%
N2	7	11.10%

**M stage**		

M0	63	100.00%
M1	–	

### Verification of the correlation between transforming growth factor-β and poly r (C) binding protein 1 antisense expression

In the 16 lung adenocarcinoma samples previously used to detect the degree of Treg infiltration and the expression of PCBP1-AS1, we also detected the mRNA expression of TGF-β, and we found that the expression of PCBP1-AS1 and TGF-β was significantly negatively correlated (Figure 11C, R = −0.519, p value = 0.0394). The expression level of TGF-β was lower in the group with high PCBP1-AS1 expression (Figure 11D). The inverse correlation between PCBP1-AS1 and TGF-β expression was also confirmed in lung adenocarcinoma cell lines (Figures 11E and 11F). Meanwhile, the related molecules in the TGF-β pathway were detected, and the TGF-β pathway was inhibited to a certain extent after the overexpression of PCBP1-AS1 (Figures 11G and 11H).

## Discussion

Lung cancer exhibits high morbidity and mortality rates and is a severe disease that threatens public health.^1^ Despite advances in lung cancer treatment, the tumors have acquired resistance to new drugs and radiotherapy. Combination therapies are commonly used in clinics to improve curative effects; however, they are accompanied by adverse reactions such as diarrhea, hypertension, and fatigue.^35^^,^^36^^,^^37^ Therefore, finding new genetic biomarkers is crucial to classifying these tumors and suggesting clinical treatment options. These new biomarkers can help researchers enhance their understanding of tumorigenesis and promote the development of the current theoretical framework of oncology.

ML is widely used in many disciplines owing to its automatic learning and accurate prediction; however, due to the invisible black-box problem, ML application in medicine is limited.^32^ In the present study, we have provided information regarding key features and tags and their roles in model development using several visual ML toolkits.

TME are mainly composed of tumor, interstitial, and immune cells and exhibit a dynamic balance between biological mechanisms unlike that shown by normal tissues.^6^ With the occurrence and growth of tumors, the composition of immune cells in TME changes, in which the levels of CD8^+^ T and NK cells decrease, whereas the levels of Tregs and regulatory B cells increase, which are beneficial phenomena for tumor growth and immune escape.^6^ Unlike immune cells that play an immunosuppressive role, Tregs play a key role in tumor survival and immune escape.^38^^,^^39^ The present study showed that Tregs played a crucial role in TME formation, and the degree of Treg infiltration was significantly correlated with the prognosis of patients with lung adenocarcinoma.

lncRNAs are involved in the genesis and development of various immune cells (immunobiology of long noncoding RNAs). Here, we screened lncRNAs related to Treg infiltration, differential lncRNAs between lung adenocarcinoma and para-cancerous tissues, and lncRNAs related to prognosis by ML, and obtained a potential biomarker, PCBP1-AS1. Enrichment analysis showed that it could regulate the cell cycle and other biological behaviors in lung adenocarcinoma. Meanwhile, behavioral experiments were performed to investigate the biological function of PCBP1-AS1 in LUAD cells. We analyzed the transcriptional regulation of PCBP1-AS1 and its possible biological function in lung adenocarcinoma by TRlnc and lncSEA.^33^^,^^34^ In addition to its known clinical significance, PCBP1-AS1 is associated with the current advances in tumor immunology, especially its relationship with Treg infiltration.

TME contains various types of cells that form a suitable environment for tumor survival and development, and various cytokines are the key factors in TME. Tumor cells interact with Tregs by secreting proteins such as TGF-β, CCL22, galectin-9, and membrane protein PD-1.^40^^,^^41^^,^^42^^,^^43^ Among them, TGF-β is critical for Treg proliferation and function, and the present results showed that PCBP1-AS1 expression was closely associated with RUNX1, which is upstream of the TGF-β pathway and controls the anergy and suppressive function of regulatory T-cells (Treg) by associating with FOXP3.^44^ We found that PCBP1-AS1 regulated the mRNA and protein expression of TGF-β and inhibited the TGF-β pathway. Based on TIDE predictions and immunotherapy cohort validation, PCBP1-AS1 can be used as a biomarker to guide clinical treatment using ICIs. In this study, a larger proportion of people who benefitted from immunotherapies belonged to the high-risk group with high PCPB1-AS1 expression. A similar result was reported by Oshi M et al., who showed that increased Treg abundance was significantly associated with ICI gene expression, which was directly related to the effectiveness of treatment with ICIs.^45^ Another study showed that immunotherapies could destroy invasive Tregs in the TME.^46^ This finding may explain the sensitivity to immunotherapy observed in patients with lung adenocarcinoma with low PCBP1-AS1 expression.

### Conclusions

In this study, Tregs were selected as key immune cells to regulate a TME, and lncRNAs related to Treg infiltration were obtained using an ML model. Combined with its role in patient prognosis and tumor-specific expression characteristics, we screened out PCBP1-AS1, and the biological regulation of PCBP1 in lung adenocarcinoma was validated. We also predicted PCBP1-AS1 regulated Tregs via the TGF-β pathway and verified them by RT-qPCR, Western blot, and immunofluorescence. Furthermore, its effect on immunotherapy responses was analyzed. We found a new biomarker and a potential therapeutic target for lung adenocarcinoma.

### Limitations of the study

At present, there are some limitations to this article. Firstly, the specific mechanism of PCBP1-AS1 regulating TGF-β is still unclear, and secondly, the regulatory effect of PCBP1-AS1 on lung adenocarcinoma has not been verified *in vivo*.

## STAR★Methods

### Key resources table

**Table:** 

REAGENT or RESOURCE	SOURCE	IDENTIFIER
**Antibodies**		

anti-GAPDH	Santa Cruz Biotechnology	Cat# sc-47724; RRID: AB_627678
anti-TGF-β	Cell Signaling Technology	Cat# 3709; RRID: AB_2063357
anti-*p*-Smad2/3	Cell Signaling Technology	Cat# 8828; RRID: AB_2631089
anti-Smad2/3	Cell Signaling Technology	Cat# 8685; RRID: AB_10889933
anti-Foxp3	Santa Cruz Biotechnology	Cat# sc-53876; RRID: AB_783444
anti-rabbit IgG	Cell Signaling Technology	Cat# 8889; RRID: AB_2716249

**Bacterial and virus strains**		

pLV-EF1a-Puro-PCBP1-AS1	KEL Biotech	N/A
pLV-EF1a-EGFP-PGK-Puro	KEL Biotech	N/A

**Biological samples**		

Human LUAD tissues and adjacent tissues	Shandong Provincial Hospital	N/A

**Chemicals, peptides, and recombinant proteins**		

Puromycin	Beyotime Biotechnology	ST551
Trizol reagent	Accurate Biotechnology	A2A0209
RPMI 1640 With L-Glutamine	VivaCell BIOSCIENCES	C3010-0500
Nutrient Mixture F-12 Ham’s	macgene	CM10025
DAPI	Beyotime Biotechnology	C1002
PI/RNase staining buffer	BD Pharmingen	550825
PMSF	Beyotime Biotechnology	ST506
formalin	Servicebio	HJ20606

**Critical commercial assays**		

BeyoClick™EdU Cell Proliferation Kit with Alexa Fluor 488	Beyotime Biotechnology	C0071
Annexin V-FITC/PI kit	YEASEN	40302
SYBR Green System	Accurate Biotechnology	A2A1436

**Deposited data**		

TCGA lung adenocarcinoma cohort	The Cancer Genome Atlas (TCGA)	https://xenabrowser.net/
GEO lung adenocarcinoma cohort	Gene Expression Omnibus (GEO)	https://www.ncbi.nlm.nih.gov/geo/
GTEx normal lung cohort	Genotype-Tissue Expression Project (GTEx)	https://xenabrowser.net/

**Experimental models: Cell lines**		

A549	Procell Life Science & Technology	CL-0016
PC-9	Procell Life Science & Technology	CL-0668

**Oligonucleotides**		

Primers for quantitative PCR: listed in Table S1	This study	N/A

**Software and algorithms**		

R version 4.1.2	R Foundation for Statistical	https://cran.r-project.org/
GraphPad Prism software version 8.0.2	GraphPad Software	https://www.graphpad.com/
ImageJ	National Institutes of Health	https://imagej.net/
FlowJo V10	BD Biosciences	https://www.flowjo.com/
SPSS (version 24.0)	IBM	https://www.ibm.com/cn-zh/spss

### Resource availability

#### Lead contact

Further information and requests for resources should be directed to the lead contact, Jiajun Du (dujiajun@sdu.edu.cn).

#### Materials availability

Plasmids in this paper will be shared freely upon request to the lead contact.

#### Data and code availability

• All data generated or analyzed during this study are included in this article. Further enquiries can be directed to the lead contact.
• This paper does not report original code.
• Any additional information required to reanalyze the data reported in this paper is available from the lead contact upon request.

### Experimental model and study participant details

#### Clinical samples

The validation cohort consisted of 63 patients from the Department of Thoracic Surgery, Shandong Provincial Hospital of Shandong Province, China. Meanwhile, 16 lung adenocarcinoma patient samples for immunofluorescence preparation were shipped from the Department of Pathology of the Shandong Provincial Hospital. Patients’ characteristics were summarized in Table 1. This study was approved by the Ethics Review Committee (SWYX: NO.2021-255) and was performed based on the Declaration of Helsinki and Good Clinical Practice guidelines defined by the International Conference on Harmonisation of Technical Requirements for Registration of Pharmaceuticals for Human Use.

#### Cell lines and cell culture

The following pulmonary adenocarcinoma cell lines were purchased for the study: The PC-9 and A549 cell lines were procured from Procell Life Science & Technology Co., Ltd (Wuhan, China). PC-9 cells were maintained in RPMI 1640 (VivaCell), A549 cells were cultured in F12K (MAC GENE). All cell lines were supplemented with 10% fetal bovine serum (Lot 1742862, Biological Industries, Israel) and 1% penicillin-streptomycin (P/S) and were incubated at 37°C with 5% CO_2_ atmosphere. The cell lines were confirmed to be free of mycoplasma contamination, and cell passaging was limited to 10 times.

### Method details

#### Construction and transfection of lentivirus

The gene sequence GenBank: NR_183167.1 of PCBP1-AS1 was integrated into pLV ncRNA-Puro-EF1A. After vector construction, A549 and PC9 cell lines were transfected with virus at moi = 10. Twelve hours after transfection, the medium was replaced with standard medium and incubation was continued for another 48 h. This was followed by a one-week screening with puromycin (2 μg/mL, provided by Beyotime Biotech).

#### Immunofluorescence

The fresh tumor tissues were isolated from resected lung adenocarcinomas during the time of surgery and then immediately fixed in formalin (Lot HJ20606, Servicebio, China). The tissues were dehydrated into wax and stored in wax blocks. The wax block was sliced into 4-mm slices using a microtome. After dewaxing and antigen retrieval, the paraffin sections were incubated with an anti-Foxp3 antibody (Santa Cruz, catalog number# sc-53876, USA) and then stained with an Alexa Fluor 594-conjugated secondary fluorescent secondary antibody (#8889S, Cell Signaling Technology, USA). The position of the nucleus was indicated by counterstaining with DAPI (#C1002, Beyotime, China). The fluorescence signal was detected with an upright fluorescence microscope (Olympus, Japan).

#### Western blot

Proteins were extracted from cells and tissues using RIPA buffer supplemented with phenylsulfonyl fluoride (PMSF) (PMSF, ST506, Beyotime Biotech, Shanghai, China) in a 100:1 ratio. For Western blotting analysis, 10% SDS-PAGE (120 V for 60 min) was used. The isolated protein bands were subsequently transferred to a polyvinylidene difluoride membrane at 100 V and maintained for 1.5 h using a Bio-Rad transfer apparatus.

The Western blotting process was then blocked with TBS/Tween 20 solution containing 5% BSA and incubated with antibodies overnight at 4° C. Bands were visualized using a ChemiDoc TM MP Imaging System (BIO-RAD, California, USA). The following primary antibodies were used in this study: GAPDH (1:500; Santa Cruz, #47724); TGF-β (1:1000; CST, #3709), *p*-SMAD2/3 (1:1000; CST, #8828) and SMAD2/3 (1:1000; CST # 8685).

#### Cell proliferation and colony formation assays

Cell proliferation was measured by sulforhodamine B (SRB) assay and cells were seeded in 96-well plates at a density of 1500 cells/well. After 24 h of culture, when cells were fully adherent, initial adherence was recorded at zero hour, and subsequent samples were collected every 24 h. Each well was fixed with 80 μL trichloroacetic acid (TCA) for at least 6 h at 4° C. After fixation, the plates were rinsed five times with tap water and dried overnight at room temperature. Subsequently, each well was stained with 0.057% (w/v) SRB solution (100 μL) for at least 30 min, followed by washing at least five times with 1% acetic acid and drying overnight at room temperature. Each well was treated with 150 μL of 10 mM Tris alkaline solution and shaken at 2000 rpm for 20 s on an IKA MS 3 digital device (Thermo Fisher, USA). The absorbance of the solution at 562 nm was measured using a microplate reader (Bio-Rad, USA).

Colony formation assays were performed by culturing at a density of 500 cells/well in a 5% CO_2_ incubator at 37° C for 2 weeks in 6-well plates. Cells in each well were fixed with paraformaldehyde for 30 min, stained with 500 μL of 0.1% crystal violet for at least 20 min, and quantified using ImageJ software.

#### EdU assay

Cells were seeded at 50% density in 12-well plates. After 24 h of culture, when cells were fully adherent, they were analyzed using the BeyoClickEdU Cell Proliferation Kit with Alexa Fluor 488 (Beyotime biotech, C0071L) according to the manufacturer’s instructions. Images were taken with a ZEISS fluorescence inverted microscope, processed with ZEN Blue Lite, and cell counting was performed using ImageJ software.

#### Wound healing assay

Monolayer cells were scratched with a 200 μL pipette tip in a 12-well plate when cell density reached 95%. Images were taken every 12 h, and the scratch area was measured using ImageJ software.

#### Transwell assay

For transwell migration assay:4 × 10^4^ cells were seeded in transwell chambers containing 200 μL serum-free medium. 600 μL of culture medium containing 20% FBS was injected into the lower culture chamber. After 24 h, cells were removed, washed with PBS, fixed with paraformaldehyde, and stained with 0.1% crystal violet. Cells from five random areas per well were photographed and counted using ImageJ software.

For transwell invasion assays, Matrigel gels were removed from −20° C and placed in a 4° C refrigerator overnight. Matrigel gel was diluted to 300 μL/mL with 4° C serum-free cell medium, and 100 μL was evenly spread on the top surface of the PET membrane in the cell culture pool. Remove and allow to dry overnight on a clean bench. 8 × 10^4^ cells were seeded in transwell chambers containing 200 μL serum-free medium. 600 μL of culture medium containing 20% FBS was injected into the lower culture chamber. After 36 h, cells were removed, washed with PBS, fixed with paraformaldehyde, and stained with 0.1% crystal violet. Cells from five random areas per well were photographed and counted using ImageJ software.

#### Annexin V-FITC/PI assay

Cells were seeded in 6-well plates at 50% density. After 24 h of culture, the cells were collected by enzyme digestion and centrifugation. Cells were washed twice with PBS, stained with Annexin V-FITC/PI kit (YEASEN, 40302), and analyzed with BD LSRFortessa and FlowJo.

#### Cell cycle analysis

Cells at 60% density were seeded in 6-well plates. After 24 h of culture, the cells were completely adherent. Cells were washed with PBS and isolated by enzymatic digestion. The enzymatic separation was terminated with medium containing FBS. The cells were centrifuged. The residue was re-suspended in 70% pre-cooled ethanol solution and fixed at −20° C for at least 4 h. The cells were centrifuged and washed twice with PBS. Cells were re-suspended in 0.5 mL PI/RNase staining buffer (550825, BD, Shanghai, China) and incubated for 15 min at room temperature. Cell cycle was determined using a Cytoflex cell analyzer and analyzed using Flowjo v10.

#### Quantitative real-time PCR assay

Total RNA was obtained with Trizol reagent (Lot A2A0209, Accurate Biotechnology, China) and quantified using the Nanodrop 2000 (Thermo Fisher Scientific, American) spectrometer. A reverse transcription kit (A2A1386) was obtained from Accurate Biotechnology. The primers targeting 18S, TGF-β and PCBP1-AS1 were obtained from Takara (Japan); their sequences are provided in Table S1. Real-time PCR was performed with the LightCycler 480 II (Roche, Swiss), and a detection kit was used along with the SYBR Green System (Lot A2A1436, Accurate Biotechnology).

#### Data preparation

The screening process of differentially expressed lncRNA was based on GTEx and TCGA databases as downloaded from University of California Santa Cruz (UCSC) Xena (https://xenabrowser.net/). In total, 1034 samples (including normal and lung adenocarcinoma) from TCGA and GTEx databases were investigated as the training cohort. Files GSE10072, GSE10245, GSE27719, GSE28571, GSE30219, GSE31210, GSE31547, GE37745, GSE40791, GSE43580, GSE50081, GSE68465, GSE72094, GSE115458, GSE83836, GSE116959, GSE32863, GSE42127, GSE41271, GSE63459, GSE75037 and GSE123352, regarding a total of 2862 samples (including lung adenocarcinoma and normal samples), were downloaded from the GEO databases as the external validation cohort.

#### Establishment and visualization analysis of the ML model

The algorithm eXtreme Gradient Boosting (XGBoost)^47^ was used to establish the model, and the area under the curve (AUC) and confusion matrix were used to test the established model. The visual analysis of the model was performed using SHapley additive exPlanations (SHAP),^48^ Partial Dependency Plot box (PDPbox),^49^ Explain Like I’m 5 (ELI5), and InterpretML.^50^ Based on cooperative game theory, an additive interpretation model was constructed using SHAP, and all features were regarded as “contributors”. The model produced a prediction value for each prediction sample, and an SHAP value was assigned to each feature in the sample.^48^ PDP graphs show the marginal effect of features on the prediction results of ML models and are used to evaluate whether the correlation between features and targets is linear, monotonous, or more complex.^49^ ELI5 is a Python package that aims to explain black-box ML models in Python. ELI5 provides weights associated with each feature to depict the importance of features in ML models. InterpretML is a Python package that automatically thermocodes and designs interactive features to provide interactive drawings to understand the prediction results.^50^

#### Analysis of differential expression of genes and construction of a prognostic model

Differently expressed lncRNAs from the combined data of GTEx and TCGA were analyzed using the R package limma (p < 0.01, Log_2_|FC| > 2). These lncRNAs were subjected to univariate and multivariate Cox regression analyses, and lncRNAs with prognostic significance were screened out. A prognostic model was constructed based on these lncRNAs with prognostic significance. Next, patients whose survival time was more than 30 days were subjected to survival analyses. The Nomogram with the calibration curve was used to construct a prognostic model, and the ROC curve showed the predictive power and accuracy of the model. A receiver operating characteristic (ROC) curve was plotted using the R package survivalROC. The C index was calculated using the R package survcomp.

#### Functional enrichment and correlation analysis

To explore functions and pathways associated with PCBP1-AS1, we firstly analyzed the expression level of PCBP1-AS1 in samples from the TCGA-LUAD dataset. The top 10% of high-expression samples and the last 10% of low-expression samples were extracted. We used the limma package and screened out the differential genes of PCBP1-AS1 in TCGA. Gene Ontology (GO) and Kyoto Encyclopedia of Genes and Genomes (KEGG) enrichment analyses were performed using the “clusterProfiler”. Moreover, Gene Set Enrichment Analysis (GSEA) was performed using the “clusterProfiler” package to explore the heterogeneity of biological processes. The gene sets “c5.go.symbols.gmt” were used for enrichment analyses. Gene Set Variation Analysis (GSVA) enrichment was performed using the hallmark gene set “c2.cp.v2023.2.Hs.symbols.gmt” extracted from the MSigDB database.

#### Immune cells infiltration analysis

QUANTISEQ, CIBERSORT-ABS and CIBERSORT algorithms were used to investigate the correlation between PCBP1-AS1 expression and a variety of immune cells such as CD4^+^ T cells, CD8^+^ T cells and regulatory T cells (Treg cells). Correlation analysis of PCBP1-AS1 and Treg cells related molecules was performed using Pearson correlation coefficient analysis.

#### Immunotherapy dataset and immunotherapy prediction

Immunotherapy datasets from three different cancer types including IMvigor210 cohort (advanced urothelial carcinoma), GSE78220 (melanoma) and GSE135222 (non-small cell lung cancer) were used to verify the prediction of PCBP1-AS1 expression on immunotherapy efficacy. According to the publishers of the data, efficacy was evaluated in the real-world immunotherapy cohorts as Response vs. non-Response or complete response (CR)/partial response (PR) vs. progressive disease (PD)/stable disease (SD). The Tumor Immune Dysfunction and Exclusion (TIDE, http://tide.dfci.harvard.edu/) Website and TIDE scores were used to predict the effect of PCBP1-AS1 on immunotherapy response in TCGA LUAD dataset.

### Quantification and statistical analysis

All R packages were run under the R 4.1.2 version. Parts of the statistical analyses were performed by GraphPad Prism (version 8.0) and SPSS 24.0 (Chicago). p < 0.05 was considered to be statistically significant. ∗p < 0.05; ∗∗p < 0.01 and ∗∗∗p < 0.001; ns, not significant.

## Supplemental information

Document S1. Figures S1 and S2 and Tables S1, S2, and S5

Table S3 Tregs infiltration related differential lncRNAs, related to Figure 3

Table S4 Differential lncRNAs between lung adenocarcinoma and normal tissues, related to Figure 3
