# Supplementary material for: Identification of key immune cells infiltrated in lung adenocarcinoma microenvironment and their related long noncoding RNA
Source: iScience. 2024 Feb 15;27(3):109220. doi: 10.1016/j.isci.2024.109220 (PMC10907860; doi:10.1016/j.isci.2024.109220)
Supplement: Document S1. Figures S1 and S2 and Tables S1, S2, and S5 [file mmc1.pdf]

iScience, Volume *27*

Supplemental information

Identification of key immune cells infiltrated in lung adenocarcinoma microenvironment and their related long noncoding RNA

Kai Wang, Tao Yan, Deyu Guo, Shijie Sun, Yong Liu, Qiang Liu, Guanghui Wang, Jingyu Chen, and Jiajun Du

Supplemental Materials

Supplementary Tables

Supplemental Table 1 Primer sequence. Related to Figure 8 and 11.

Primer sequence	
Name	Sequence (5'-3')
PCBP1-AS1-F	GACGATAGAGATCAGTGGGCTTTG
PCBP1-AS1-R	TGCTGTCTGAGAATGCTGATGG
18SrRNA-F	AAACGGCTACCACATCCAAG
18SrRNA-R	CCTCCAATGGATCCTCGTTA
TGF-β-F	TACCTGAACCCGTGTTGCTCTC
TGF-β-R	GTTGCTGAGGTATCGCCAGGAA

Supplemental Table 2 3896 sample groups. Related to Figure 1-2.

Group	Type	Composition	Normal	Tumor	Total
group1	train	TCGA-LUAD; GTEx-Lung	443	591	1034
group2	test	GSE116959	11	52	63
group3	test	GSE32863	54	47	101
group4	test	GSE10072	49	58	107
group5	test	GSE31210	20	225	245
group6	test	GSE63459	29	28	57
group7	test	GSE75037	71	62	133
group8	test	GSE40791	98	85	183
group9	test	GSE30219; GSE72094	14	512	526
group10	test	GSE68465	19	443	462
group11	test	GSE10245; GSE28571; GSE31547; GSE37745; GSE42127; GSE50081	20	459	479
group12	test	GSE27719; GSE115458; GSE83836; GSE43580; GSE41271; GSE123352	174	332	506

Supplemental Table 5 lncRNAs included in Multivariate Cox regression analyses. Related to Figure 3.

id	HR	HR.95L	HR.95H	pvalue
PCBP1-AS1	0.532361	0.263473	1.075664	0.078965
AC125494.1	5.497964	0.908768	33.26217	0.063482
AC125611.3	1248.967	5.422766	287661.1	0.010195
AC099850.3	1.160915	0.984346	1.369155	0.076306
AC010976.2	0.768169	0.414346	1.424133	0.40237
FENDRR	0.960531	0.6655	1.386356	0.8297
AC133550.1	37.55361	0.14649	9627.107	0.200116
VIPR1-AS1	0.640648	0.264532	1.55153	0.323806

Supplemental Figures

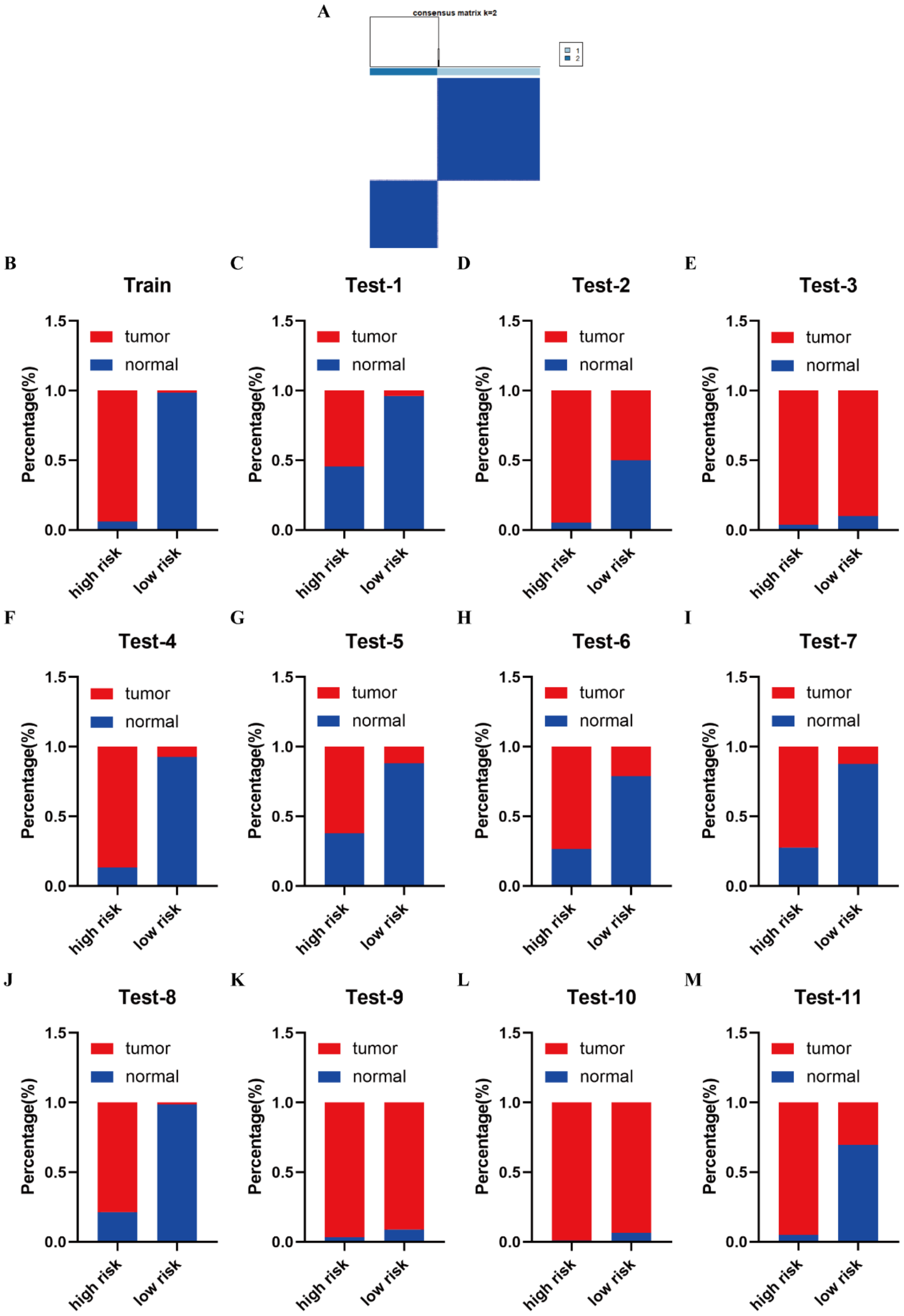

Supplemental Figure 1 SHAP model predicts tumor proportion. Related to Figure 1-2. **(A)** Results of PCA clustering. **(B)** The proportion of tumors in the high and low risk groups in the training set. **(C-M)** The proportion of tumors in the high and low risk groups in the testing sets.

A

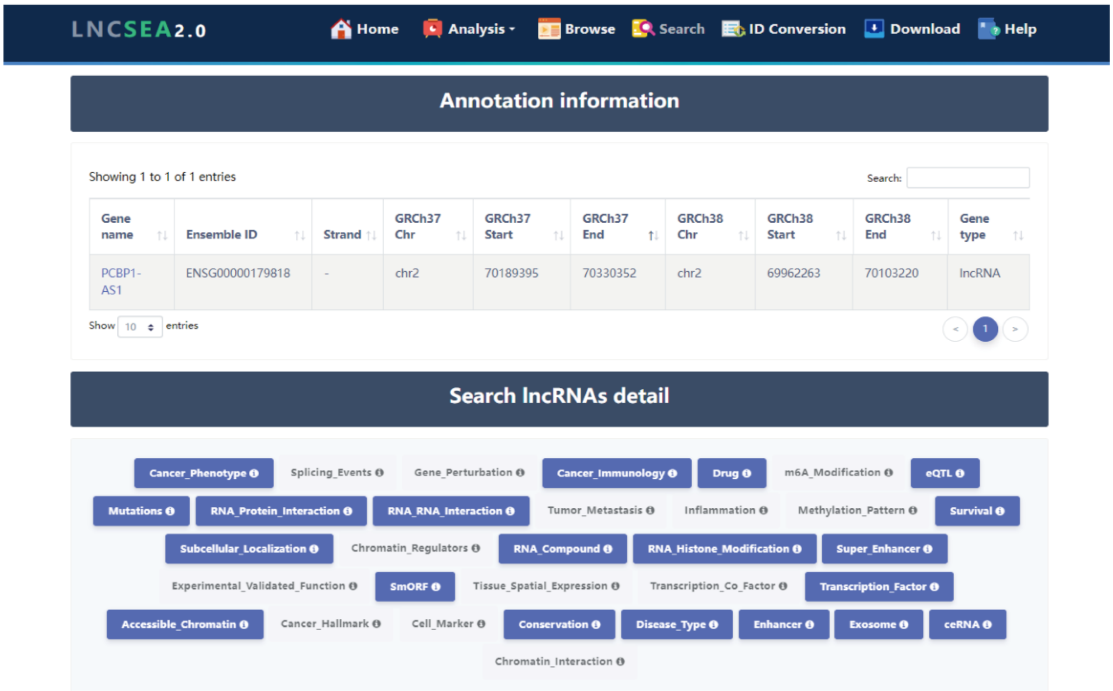

Supplemental Figure 2 Results of lncSEA analysis. Related to Figure 4. **(A)** LncSEA shows the results of PCBP1-AS1.
